# PCB 126 and Other Dioxin-Like PCBs Specifically Suppress Hepatic PEPCK Expression via the Aryl Hydrocarbon Receptor

**DOI:** 10.1371/journal.pone.0037103

**Published:** 2012-05-16

**Authors:** Wenshuo Zhang, Robert M. Sargis, Paul A. Volden, Christopher M. Carmean, Xiao J. Sun, Matthew J. Brady

**Affiliations:** 1 Department of Medicine, Kovler Center for Biomedical Discovery, The University of Chicago, Chicago, Illinois, United States of America; 2 Department of Medicine, University of Maryland School of Medicine, Baltimore, Maryland, United States of America; Johns Hopkins University, United States of America

## Abstract

Dioxins and dioxin-like compounds encompass a group of structurally related heterocyclic compounds that bind to and activate the aryl hydrocarbon receptor (AhR). The prototypical dioxin is 2,3,7,8-tetrachlorodibenzo-*p*-dioxin (TCDD), a highly toxic industrial byproduct that incites numerous adverse physiological effects. Global commercial production of the structurally similar polychlorinated biphenyls (PCBs), however, commenced early in the 20^th^ century and continued for decades; dioxin-like PCBs therefore contribute significantly to total dioxin-associated toxicity. In this study, PCB 126, the most potent dioxin-like PCB, was evaluated with respect to its direct effects on hepatic glucose metabolism using primary mouse hepatocytes. Overnight treatment with PCB 126 reduced hepatic glycogen stores in a dose-dependent manner. Additionally, PCB 126 suppressed forskolin-stimulated gluconeogenesis from lactate. These effects were independent of acute toxicity, as PCB 126 did not increase lactate dehydrogenase release nor affect lipid metabolism or total intracellular ATP. Interestingly, provision of cells with glycerol instead of lactate as the carbon source completely restored hepatic glucose production, indicating specific impairment in the distal arm of gluconeogenesis. In concordance with this finding, PCB 126 blunted the forskolin-stimulated increase in phosphoenolpyruvate carboxykinase (PEPCK) mRNA levels without affecting glucose-6-phosphatase expression. Myricetin, a putative competitive AhR antagonist, reversed the suppression of PEPCK induction by PCB 126. Furthermore, other dioxin-like PCBs demonstrated similar effects on PEPCK expression in parallel with their ability to activate AhR. It therefore appears that AhR activation mediates the suppression of PEPCK expression by dioxin-like PCBs, suggesting a role for these pollutants as disruptors of energy metabolism.

## Introduction

PCBs are a group of bicyclic chlorinated hydrocarbons that are lipophilic and highly resistant to physical, chemical, and enzymatic breakdown [1]. PCBs saw widespread industrial use in the United States from the 1930s until their production was restricted in 1979 under the Toxic Substances Control Act; the Stockholm Convention on Persistent Organic Pollutants in 2001 banned PCB production internationally. However, due to seepage into the environment, accidental spills, and improper disposal, combined with their persistence and propensity for bioaccumulation, PCBs are still found at measurable levels in soil [2], fresh water [3], aquatic wildlife [4], and mammals, including humans [5]. Exposure to PCBs and other dioxins and dioxin-like compounds incites a variety of adverse neurological, reproductive, developmental, immunological, and metabolic effects in both wildlife and humans depending on dose, timing, and length of exposure [1].

PCBs comprise two categories: dioxin-like and non-dioxin-like, depending on their structural and chemical properties as well as their physiological impact. The term dioxin-like refers to structural and physiological similarities to the prototypical dioxin TCDD, a highly toxic byproduct of certain industrial processes. TCDD and its congeners, including dioxin-like PCBs, are thought to exert their toxic effects primarily through activation of the aryl hydrocarbon receptor (AhR), a widely expressed nuclear transcription factor that binds a broad range of xenobiotics [6]. Unliganded AhR resides in the cytosol; binding of a productive ligand induces translocation into the nucleus, where AhR dimerizes with the aryl hydrocarbon receptor nuclear translocator, binds to xenobiotic response elements of its target genes, and induces transcription. The actual downstream targets of activated AhR remain largely unknown, but include several members of the cytochrome P450 (CYP) family of first-phase drug metabolizing enzymes. The putative mechanism of dioxin-induced toxicity is a combination of high specificity for AhR binding and persistent AhR activation secondary to poor metabolism of dioxins by the CYPs, leading to a range of organ- and species-dependent maladaptive responses (reviewed in [7]).

Productive AhR binding by a putative ligand is a key requisite for assignment of a toxic equivalence factor (TEF). The TEF is an index developed in the 1980s to compare the relative toxicity of dioxins and their congeners, and uses TCDD as its reference standard (TEF = 1). The World Health Organization stipulates that a compound must bear structural similarity to TCDD, bind to and activate the AhR, and persist and bioaccumulate in the food chain [1] to be assigned a TEF. PCB 126 is a dioxin-like PCB with the highest TEF amongst the PCBs (TEF = 0.1); the next most potent PCB congener, PCB 169, has a TEF more than three-fold lower [8]. Despite its relatively minor contribution as a constituent of PCB mixtures by weight, the potency of PCB 126 underlies its toxicological significance as the major contributor to the toxic equivalent in common PCB mixtures, which may be as high as 90% [9], [10]. In humans, a wide range of dioxin-like compounds can be detected, but the majority of toxicity is attributed to only seven species, one of which is PCB 126 [5].

The majority of studies pertaining to the metabolic ramifications of dioxin exposure use animal models, mostly rodents [11], [12], [13] — although sensitivity to the wasting effect of dioxins demonstrates broad strain [11], [14] and species [15], [16], [17] specificity. Coincident observations across such studies include impaired gluconeogenesis and, more specifically, reductions in the activity, protein level, and gene expression of PEPCK [14], [18], [19], [20], [21]. Given the broader distribution of, and environmental contamination by, PCBs, compared to TCDD, the effects of PCB 126 on hepatic glucose metabolism are potentially important. To date, few studies have characterized the effects of PCB 126 on mammalian metabolism [21], [22] nor the impact of PCB 126 on hepatic gluconeogenesis in a tightly controlled *in vitro* system. While in vivo studies have inherent advantages with respect to physiological relevance, *in vitro* systems are arguably optimally suited for examining the direct effects of PCB 126 in the liver by avoiding potentially confounding indirect/secondary effects. For example, enhancement of lipolysis in adipose tissue —secondary, perhaps, to dioxin-induced anorexia [13]—and the resulting increase in hepatic FFA delivery could affect HGO in vivo, whether via substrate-level and/or hormonal mechanisms [23], [24]. The present study, therefore, utilizes primary mouse hepatocytes to address the direct metabolic impact of PCB 126 and the role of the AhR as a mediator of the effects of dioxin-like PCBs on hepatic glucose metabolism, with particular emphasis on gluconeogenesis.

## Materials and Methods

### Ethics Statement

The research presented in this manuscript was conducted using protocols approved by IACUC at the University of Chicago.

### Materials

All PCBs were from AccuStandard (New Haven, CT). Unless mentioned otherwise, reagents were from Sigma. For a list of PCB congeners tested, please refer to Table 1.

**Table 1:** Summary of compounds.

Compound	CAS	Common name	Class	TEF*
PCB 126	57465-28-8	3,3′,3,3′,5-pentachlorobiphenyl	Non-ortho-substituted coplanar	0.1
PCB 169	32774-16-6	3,3′,4,4′,5,5′-hexachlorobiphenyl	Non-ortho-substituted coplanar	0.03
PCB 81	70362-50-4	3,4,4′,5-tetrachlorobiphenyl	Non-ortho-substituted coplanar	0.0003
PCB 77	32598-13-3	3,3′,4,4′-tetrachlorobiphenyl	Non-ortho-substituted coplanar	0.0001
PCB 105	32598-14-4	2,3,3′,4,4′-pentachlorobiphenyl	Mono-ortho-substituted coplanar	0.00003
PCB 153	35065-27-1	2,2′,4,4′,5,5′-hexachlorobiphenyl	Di-ortho-substituted non-coplanar	N/A
Myricetin	529-44-2	3,3′,4,5,5′,7-hexahydroxyflavone	Flavonol	N/A

* TEF values are based on WHO 2005 published values.

### Isolation and culture of primary mouse hepatocytes

A modification of the non-recirculating two-step perfusion method as detailed in [25] was used. Eight to twelve week-old male C57BL/6 mice were anesthetized with isoflurane and the portal vein was cannulated with a twenty-three gauge needle. Upon successful cannulation, the inferior vena cava (IVC) was immediately cut to allow fluid to drain. Hank's Balanced Salt Solution (HBSS; Invitrogen) containing 5 mM glucose supplemented with 0.5 mM EGTA and 25 mM HEPES (pH 7.4 at 37°C) was perfused at 9 mL/min for 6 min with periodic clamping (5 s clamp every 30 s) of the IVC to accelerate the process. DMEM containing 5 mM glucose (Mediatech) supplemented with 100 U/mL Penicillin and 0.1 mg/mL Streptomycin (Pen/Strep), 15 mM HEPES, and 100 U/mL of collagenase (Type IV, Worthington) was then perfused at 9 mL/min for an additional 6 to 8 min to digest the liver. Intermittent clamping of the IVC was also performed during this step of the process to augment total cell yield. After sufficient digestion, the gall bladder was removed and the liver was excised and transferred to a 9.5-cm Media-Miser dish (Fisher) containing 15 mL of the same medium used for digestion. Cells were liberated by tearing and shaking of the liver with forceps followed by gentle trituration. The cell suspension was then filtered through a 74 µm stainless steel strainer (Dual Manufacturing), washed 3 times by spinning at 50×*g* for 2 minutes at 4°C, and resuspended in isolation medium (DMEM with 25 mM glucose supplemented with Pen/Strep, 15 mM HEPES, 100 nM dexamethasone and 10% FBS). Viability and yield were assessed by counting cells that excluded trypan blue; viability was >90% for all preparations, with an average viable yield of 4×10^7^ cells per animal. Hepatocytes were plated on collagen-coated (5 µg/cm^2^ Type I collagen; BD) 12 or 24-well plates at an initial density of 65–70% to attain a confluent monolayer the following day. Cells were allowed to attach for 1 h at 37°C in a humidified 5% CO_2_ incubator, washed once with DMEM (5 mM glucose), and the media then changed to DMEM (5 mM glucose) supplemented with Pen/Strep, 5 mM HEPES, 10 nM dexamethasone, and 10% FBS. Media was changed 3 h later to serum-free, phenol red-free DMEM (Mediatech) supplemented with 5 mM glucose, 44 mM NaHCO_3_, 2 mM L-glutamine, Pen/Strep, 5 mM HEPES (pH 7.4), and 10 nM dexamethasone for overnight culture/treatment. All cell preparations were used within 30 h of isolation. Additional details, images, and videos pertaining to primary hepatocyte isolation and culture may be found at the primary author's personal protocol website: www.mouselivercells.com.

### Treatment of cells

Cells were treated at the time and in the media as described above. For inhibitor studies, cells were pre-incubated as indicated for 1 h prior to addition of PCBs; all PCB incubations were 16 h in length unless explicitly noted otherwise. Forskolin stimulation for gene expression studies was performed for 3 h at a final concentration of 25 µM after direct addition to cells at h 13. All compounds and inhibitors were prepared in DMSO; final DMSO concentration for treatments and vehicle controls were identical and ranged from 0.35–0.75%.

### Total glycogen determination

Glycogen was measured by combination and extensive modifications of previous methods [26], [27]. Following treatment, the media was removed and cells were washed twice with cold PBS. Cells were lysed with 0.75% SDS and lysates transferred into microfuge tubes. A small aliquot was removed for protein determination (Pierce), and protein was precipitated for 1 h at 4°C from the remainder of the lysate by addition of 100% TCA to a final concentration of 5–10%. TCA lysates were spun at 4°C at 14,000×*g* for 10 min, and 2.5 volumes of 95% EtOH were added to the supernatant. Glycogen was allowed to precipitate at −80°C for >1 h, pelleted by spinning at 14,000×*g* for 20 min at 4°C, washed with 3 volumes of 70% EtOH, re-spun, and dried using a SpeedVac. Glycogen was digested using glucoamylase in 0.2 N sodium acetate (pH 4.4–4.6 at room temperature), and the digest was neutralized to pH 7.0 with NaOH just prior to glucose quantification. Liberated glucose was assayed via the glucose oxidase-peroxidase method by measuring the oxidation of 2,2′-azino-bis(3-ethylbenzthiazoline-6-sulphonic acid; ABTS) at 405 nm (Thermo Multiskan MCC). Conversion of absorbance to µg glucose was performed against a glucose standard curve.

### Gluconeogenesis assay

Gluconeogenesis was performed in cells after 16 h of treatment. Briefly, cells were washed twice with glucose-free, phenol red-free DMEM. The assay was performed in glucose-free, phenol red-free media supplemented with 44 mM NaHCO_3_, 2 mM L-glutamine, Pen/Strep, 10 mM HEPES (pH 7.4), 10 nM dexamethasone, and 10 mM of the specified substrate; 25 µM forskolin was also added to the appropriate media stocks. At 1 h intervals, an aliquot of media was removed for analysis of glucose by the glucose oxidase-peroxidase assay as described for glycogen determination. The remainder of the media was then removed and fresh media added for the next time point. At the termination of the assay, cells were lysed in 0.75% SDS for measurement of protein (BCA).

### Lactate dehydrogenase (LDH assay)

LDH in the supernatant was measured using a kit (Cayman Chemical) with slight modifications to the manufacturer's instructions. Total LDH was measured after removal of all media, washing cells twice with cold PBS, and releasing intracellular LDH by hypotonic lysis (60 min with shaking at 4°C in the dark in H_2_0 buffered with 5 mM HEPES). Absorbance of the reduced tetrazolium salt, INT, was measured at 490 nm on a spectrophotometer (Molecular Devices Versamax). Conversion of luminescence to LDH activity was performed against an LDH standard.

### Ethoxyresorufin-O-deethylase (EROD)

The conversion of 7-ethoxyresorufin (7-ER) to resorufin was performed by modification of a previous method [28]. Cells were washed twice in cold PBS, dried quickly by vigorous shaking, and disrupted by a single round of freezing/thawing (−80°C/37°C). Thawed plates were placed on ice and the EROD assay mixture was added (1 mg/mL BSA, 5 µM 7-ER, and 0.5 mM NADPH in 50 mM Tris (pH 7.4 at 37°C)). The reaction was initiated by placing plates on a shaker at 37°C in the dark. Total incubation time was 15 min, and the assay was terminated by addition of 0.8 volumes of 2 M glycine (pH 10.4 at room temperature). The terminated reaction mix was transferred into microfuge tubes, spun for 1 min at 14,000×*g* at room temperature, and an aliquot of the supernatant was transferred to a solid black fluorescence microwell plate. Fluorescence of resorufin was assessed on a fluorescence plate reader (Beckman Coulter DTX 880) at 535 nm excitation/595 nm emission. Conversion of fluorescence units to ng of resorufin was performed against a resorufin standard curve.

### Quantitative real-time PCR (qRT-PCR)

RNA was isolated using the Qiagen RNeasy kit. Briefly, all media was removed and cells were rapidly washed twice with cold PBS. Buffer RLT containing 10 µL/mL β-mercaptoethanol was added to all wells, and plates were stored immediately at −80°C for later processing. The purity and concentration of RNA was assessed using a NanoDrop 2000; 260/230 ratios were consistently >2.0. Synthesis of cDNA was performed with 0.5 µg of RNA using the Bio-Rad iScript kit, and was stored at −20°C. Quantitative RT-PCR was performed using Sybr Green (Bio-Rad) on a Bio-Rad MyiQ real-time PCR detection system. Primers (Table S1) were from IDT and were designed using PerlPrimer v1.1.18; when possible, primer pairs spanned introns. Primer specificity was assessed by melt curve analysis and agarose gel evaluation. Gene expression levels were evaluated by the delta-delta Ct method after confirmation that amplification efficiency was between 90%–110% for all primer pairs. Ribosomal 18S was used as a reference gene to control for total mRNA recovery, and the suitability of 18S as a reference gene was confirmed by validation against beta-actin expression.

### Beta oxidation

Oxidation of palmitate was determined by measurement of tritiated H_2_0 released during beta oxidation of [9, 10-^3^H]-palmitic acid, based on extensive modification of a previous method [29]. A concentrated solution of BSA-coupled palmitate was made by dissolving sodium palmitate in 0.01 N NaOH and adding [9, 10-^3^H]-palmitic acid (Perkin-Elmer) in a 70°C water bath. The labeled mixture was coupled to 2 mM BSA (Jackson ImmunoResearch) in HBSS to reach a final BSA-to-palmitate molar ratio of 3∶1. Immediately before addition of the BSA-palmitate concentrate, media was removed from wells and replaced with HBSS. The BSA-palmitate concentrate was then diluted into HBSS already present in wells to attain the desired palmitate concentration (200 µM). The reaction was allowed to proceed for 1 h, after which media was collected, protein precipitated with TCA, and the clarified supernatant alkalinized with 6 N NaOH; replicate aliquots of media (specificity standards) that had never been applied to cells were processed in the same manner to assess radioactivity not attributable to beta oxidation. Alkalinized samples were applied to 1 mL columns containing Dowex 1×2–400 ion-exchange resin, eluted with water, and the flow-through counted after addition of scintillation fluid (Beckman Coulter LS6500); counts from specificity standards were subtracted from total eluted counts. When used, L-carnitine was added concurrently with the substrate, while etomoxir was preincubated for 30 min prior to the start of beta oxidation.

### Lipogenesis

Lipogenesis from glucose was determined by measuring the incorporation of [^14^C(U)]-glucose (American Radiolabeled Chemicals) into the lipid extractable fraction of cell lysates. Cells were incubated with at least 1.5 µCi of ^14^C glucose per well containing the indicated concentration of glucose, and the assay was allowed to proceed for the specified time; if used, insulin was added in conjunction with radiolabel. The reaction was terminated by removal of all media followed by three washes with cold PBS. Cells were hypotonically lysed using H_2_0 (300 µL/well in a 12-well plate) with gentle rocking at room temperature and then scraped into 6 mL plastic scintillation vials (PerkinElmer). One additional volume of H_2_0 was added to wells to facilitate transfer of residual lysate. Separation of lipid soluble radioactivity was performed by addition of the maximum volume of organic scintillation fluid (Betafluor) that still allowed for enough air space to permit shaking (∼4.5 mL). Samples were vigorously shaken and allowed to sit for >4 h; media ^14^C standards were processed in the same manner to ensure specificity of partitioning, and partitioning blanks were made for ^14^C standard counting. After partitioning, the organic phase was carefully transferred to new scintillation vials and counted (Beckman Coulter LS6500). ^14^C media standards were added to partitioning blanks for conversion of counts per minute into µg of glucose incorporated. Due to the limited ability of even small volumes of aqueous liquids to remain dispersed in the organic scintillation fluid, standards were shaken well and counted immediately in replicate.

### ATP

ATP was measured using a commercial luminescence kit from total lysate with slight modifications to the manufacturer's instructions (Roche CLS II, #1-699-695). All readings were performed in 96-well format using a luminescence-capable plate reader (Beckman Coulter DTX 880). Conversion of luminescence to ng ATP was performed using an ATP standard curve.

### Statistical analysis

Comparisons between two treatments or conditions were performed with two-tailed Student's *t*-tests. Comparisons between >2 treatments or conditions were made using one-way ANOVA with post-hoc Tukey-Kramer analysis after confirmation of dataset normality. SAS JMP 7.0 was used for all analyses, and significance was set at *p*<0.05.

## Results

### PCB126 reduces basal glycogen content in primary mouse hepatocytes

**Figure 1:**
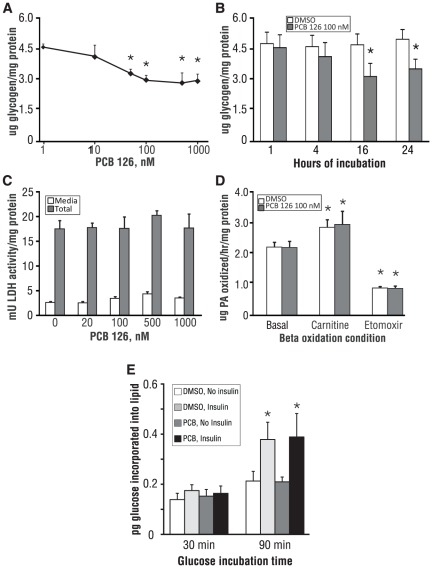
Preliminary validation of PCB effects on metabolic functions and toxicity in primary mouse hepatocytes. (A) Suppression of total intracellular glycogen by increasing doses of PCB 126; *p<0.05 vs. DMSO control. (B) Maximum suppression of total intracellular glycogen at 100 nM PCB 126 after 16 h of incubation; *p<0.05 vs. DMSO control. (C) PCB 126 is not cytotoxic at up to 1 mM, and also does not affect cellular expression of LDH. (D) PCB 126 has no effect on mitochondrial beta oxidation of palmitic acid; *p<0.05 vs. DMSO for each respective beta oxidation condition. (E) PCB 126 has no effect on basal or insulin-stimulated lipogenesis; *p<0.05 for insulin effect vs. respective no-insulin control. Unless otherwise noted, all PCB treatments were 16 h in length. Data are average of 3 experiments; error bars are −/+ SD.

Previous studies focused on the effect of dioxin-like PCBs on total glycogen content comprise feeding and/or exposure experiments performed mainly in non-mammalian aquatic animals [30] and only rarely in mammals [31]. To directly determine the effect of dioxin-like PCBs on hepatic glycogen levels, primary mouse hepatocytes were incubated with varying doses of PCB 126 for 16 h; PCB 126 was selected as the reference PCB for this study due to its TEF (highest amongst PCBs). PCB 126 caused dose-and time-dependent decreases in glycogen stores, which were maximal at 100 nM and 12–18 h, respectively; no further reduction was seen at concentrations up to ten-fold higher or for treatment periods beyond 18 h (**Figure 1A–B**). For comparison, PCB 77, a structurally similar dioxin-like PCB with a TEF several orders of magnitude lower than that of PCB 126, as well as PCB 153, a non-dioxin-like PCB with no documented TEF [8], were tested. Neither PCB 77 nor PCB 153 significantly affected cellular glycogen levels within the aforementioned concentration and duration parameters, although PCB 77 trended towards significance (*p*<0.10) (**Figure S1**).

To confirm that the effect of PCB 126 on glycogen was not secondary to acute toxicity, lactate dehydrogenase (LDH) release into the media relative to total intracellular LDH was measured (**Figure 1C**). PCB 126 had no effect on total or released LDH at concentrations up to 1 mM (10-fold higher than the maximally effective dose of 100 nM). Viability was additionally assessed by measuring intracellular ATP levels, which were unchanged (**Figure S2**). Glycogen levels in cultured primary murine hepatocytes tend to change inversely with respect to the rate of beta-oxidation and lipogenesis (unpublished observations), and others have reported that dioxin-like PCBs and TCDD augment hepatic lipogenesis [21], [32]. However, in the current system, PCB 126 had no effect on beta-oxidation of long-chain fatty acids (LCFA; palmitate used in this study) under basal conditions (**Figure 1D**). Additionally, PCB 126 did not reduce the stimulation of beta-oxidation by carnitine, which increases mitochondrial import of LCFAs, nor did PCB 126 affect the suppression of beta-oxidation by etomoxir, which blocks mitochondrial uptake of LCFAs (**Figure 1D**). PCB 126 also had no effect on basal or insulin-stimulated lipogenesis from glucose (**Figure 1E**), indicating that this agent does not interfere with lipid metabolism in cultured hepatocytes under the stated conditions.

### PCB 126 selectively suppresses forskolin-stimulated gluconeogenesis from lactate but not glycerol

**Figure 2:**
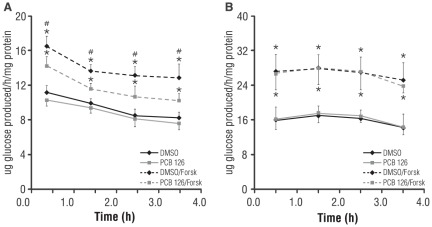
Differential impact of PCB 126 forskolin stimulated hepatic GNG with lactate vs. glycerol. (A) With 10 mM lactate as the carbon source, PCB 126 (100 nM) has no effect on basal GNG, but suppresses forskolin-stimulated glucose output. (B) With 10 mM glycerol as the carbon source, PCB 126 has no effect on either basal or forskolin-stimulated GNG, although total GNG is approximately 2-fold higher for all conditions. *p<0.05 for forskolin effect relative to respective −/+ PCB condition, #p<0.05 for forskolin effect in DMSO vs. PCB condition. All PCB treatments were 16 h in length and forskolin (25 µM) was added at the start of the GNG assay as described in Materials and Methods. Data are average of 3 experiments; error bars are −/+ SD.

In the liver, 50% or more of total glycogen arises from the indirect pathway, where non-glucose precursors provide the carbon backbone for glycogen via gluconeogenesis (GNG) [33]. Therefore, the impact of PCB 126 on GNG was evaluated by monitoring the conversion of lactate to glucose. Sixteen hour pretreatment with PCB 126 did not affect the basal (unstimulated) rate of GNG. Treatment of cells with the adenylyl cyclase activator, forskolin, significantly increased GNG in control hepatocytes, yet this response was markedly reduced by PCB 126 exposure (**Figure 2A**).

Suppression was evident during the first hour of forskolin stimulation, and persisted throughout the entire four-hour time course used for the assay. The contribution of glycogen (due to glycogenolysis of existing stores) to total glucose output in the forskolin-stimulated condition was negligible, based on the fact that 25 µM forskolin induces near-complete glycogenolysis within 30 minutes under the conditions used in these 4 h experiments (data not shown). Inclusion of 100 nM PCB 126 only during the 4 h GNG assay had no effect on basal or forskolin-stimulated GNG, indicating that PCB 126 was not exerting its effects via allosteric inhibition of GNG enzymes (**Figure S3**). Together, these results suggested that PCB 126 was suppressing GNG primarily by blunting forskolin-stimulated gluconeogenic gene expression. When glycerol was used as a carbon source in place of lactate, there was an increase in both basal and forskolin-stimulated GNG (**Figure 2B** (glycerol) vs. **Figure 2A** (lactate), note y-axis units). More interestingly, substitution of lactate with glycerol as a GNG substrate prevented PCB 126 from suppressing forskolin-stimulated GNG (**Figure 2B**). Thus, acute PCB 126 exposure specifically suppresses hepatic GNG in a substrate-dependent manner, likely at the level of GNG gene transcription.

### PCB 126 selectively suppresses forskolin induction of PEPCK, but not G6pc

GNG is controlled by the expression of several rate-limiting enzymes, primarily phosphoenolpyruvate carboxykinase (PEPCK) and glucose-6-phosphatase (G6pc). Several previous studies have shown that TCDD suppresses the activity and/or expression of PEPCK [11], [12], [13], [19], [20], [34], and when measured, the activity of G6pc decreased more modestly and was subsequent to the effects of TCDD on PEPCK [13], [14], [18], [19]. To our knowledge, only a single paper has reported that PCBs decrease PEPCK expression [21], but the molecular mechanism by which PEPCK mRNA levels were lowered, and the physiological impact on hepatic metabolism were not investigated.

**Figure 3:**
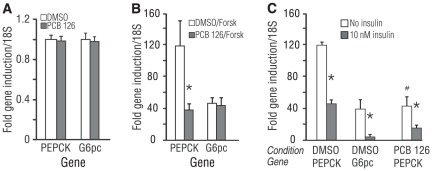
Differential impact of PCB 126 on key forskolin-responsive gluconeogenic genes. (A) PCB 126 has no effect on basal PEPCK or G6Pc expression. (B) PCB 126 selectively suppresses forskolin induction of PEPCK gene expression, but not that of G6pc. (C) Insulin strongly suppresses forskolin induction of PEPCK, and the effect of PCB 126 is additive with that of insulin; *p<0.05 for insulin effect within each condition and gene, #p<0.05 for PCB 126 effect on PEPCK induction independent of insulin. All PCB 126 treatments were 16 h in length while forskolin (25 µM) and insulin (10 nM) were added at the start of the assay. Data are average of 3 experiments for panels A–B, and 2 experiments for panel C; error bars are −/+ SD.

To elucidate the mechanism for the suppression of forskolin-stimulated GNG with lactate but not glycerol, the effect of short-term forskolin stimulation on gluconeogenic gene expression in PCB 126-treated hepatocytes was evaluated using qRT-PCR. Consistent with the GNG data, 16 h PCB 126 treatment did not alter the expression of PEPCK mRNA in the basal (non-forskolin stimulated) state (**Figure 3A** **, Left**). Three hours of 25 µM forskolin treatment robustly augmented PEPCK transcription in the vehicle-treated control, while PCB 126 treatment blunted this response by more than 65% (**Figure 3B** **, Left**). In parallel, the regulation of G6pc gene expression by PCB 126 in the absence and presence of forskolin was determined. Similar to PEPCK, basal levels of G6pc were unchanged by PCB 126 (**Figure 3A** **, Right**). G6pc gene transcription was also strongly upregulated by forskolin, but unlike PEPCK, PCB 126 was unable to blunt the induction of G6pc by forskolin (**Figure 3B** **, Right**). The impact of PCB 126 and forskolin on the expression of pyruvate carboxylase (Pcx) and fructose-1,6-bisphosphatase (Fbp1), two other key GNG genes, was also assessed. Neither gene was significantly induced by 3 h of 25 µM forskolin, nor did PCB 126 have any effect in the absence or presence of forskolin (data not shown).

Insulin is the principle means by which PEPCK and G6pc are hormonally suppressed (reviewed in [35]). To investigate the specificity of PCB 126 on PEPCK expression, 10 nM insulin was co-incubated with forskolin, and the induction of PEPCK and G6pc assessed by qRT-PCR. Insulin did not affect the basal expression of PEPCK or G6pc (data not shown). However, insulin suppressed forskolin-driven PEPCK gene expression by approximately 65% (**Figure 3C** **, Left**). Unlike PCB 126, insulin potently suppressed forskolin-stimulated G6pc expression as well (**Figure 3C** **, Middle**). Moreover, the suppressive effect of insulin was additive to that of PCB 126 with respect to PEPCK (**Figure 3C** **, Right**), resulting in an additional 65% suppression of PEPCK induction by forskolin. PCB 126 had no effect on other insulin-responsive metrics (**Figure 1E**), implying a non-insulin-related mechanism for PCB 126 specific to suppression of forskolin-stimulated PEPCK mRNA.

### AhR mediates PCB 126 suppression of forskolin-stimulated PEPCK gene expression

**Figure 4:**
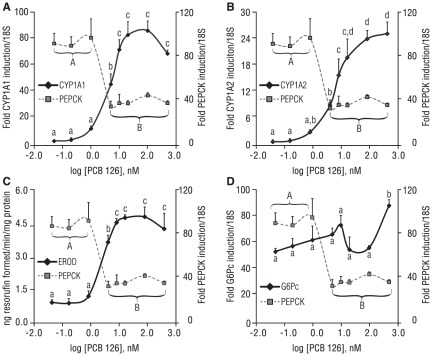
Interaction of CYP1A1, CYP1A2, EROD, and G6Pc versus PEPCK gene expression with PCB 126 dose-response. (A) EC_50_ of PCB 126 for CYP1A1gene expression and suppression of forskolin induction of PEPCK gene expression closely correlate. (B) EC_50_ of PCB 126 for CYP1A2 gene expression and suppression of forskolin induction of PEPCK gene expression are modestly correlated but demonstrate the expected directionality. (C) EC_50_ of PCB 126 for EROD activity induction and suppression of forskolin induction of PEPCK gene expression closely correlate. (D) Forskolin-induced G6pc gene expression fails to demonstrate a clear relationship with dose-dependent effect of PCB 126 on forskolin-induced PEPCK gene expression. Tested concentrations of PCB 126 in base 10 format are: 0.05, 0.20 1.0, 5.0, 10.0, 20.0, 100.0, and 500.0. Alphabetical letters separate doses of PCB 126 that elicited significantly (p<0.05) different degrees of response for the parameter designated on the left-hand y-axis (lowercase) or right-hand y-axis (uppercase). Data are average of 3 experiments; error bars are −/+ SD.

The AhR is the classic target of dioxins, although several studies have postulated that the effect of TCDD on PEPCK is not mediated by the AhR [14], [18], [20]. To evaluate the contribution of AhR to our findings in primary mouse hepatocytes, the effects of PCB 126 on gluconeogenic gene expression (PEPCK and G6pc) and downstream AhR targets was examined. CYP1A1 and CYP1A2 gene induction, as well as augmentation of EROD activity, were used as readouts for AhR agonism. Concurrently, forskolin stimulation of PEPCK and G6Pc gene expression was compared against PCB 126 concentration. Consistent with AhR activation, PCB 126 dose-dependently increased transcription of CYP1A1 (**Figure 4A**) and CYP1A2 (**Figure 4B**) and augmented EROD activity as well (**Figure 4C**). The three lowest PCB 126 doses, namely 0.05 nM, 0.20 nM, and 1.0 nM, all failed to measurably activate AhR or suppress forskolin-induced PEPCK expression in a statistically significant manner; however, the next-highest dose of PCB 126, 5 nM, significantly activated AhR by all three parameters, and also markedly suppressed forskolin-induced PEPCK expression (**Figures 4A–C**). There was no clear relationship between the concentration of PCB 126, AhR activation, and forskolin-stimulated G6pc expression (**Figure 4D**), supporting a specific effect of PCB 126 towards PEPCK. Forskolin-stimulated G6pc mRNA demonstrated some variance across PCB 126 concentrations tested, but these differences failed to achieve statistical significance (**Figure 4D**). Forskolin (25 µM, 3h) had no measurable effect on CYP1A1, CYP1A2, or EROD by any tested concentration of PCB 126 (data not shown).

**Figure 5:**
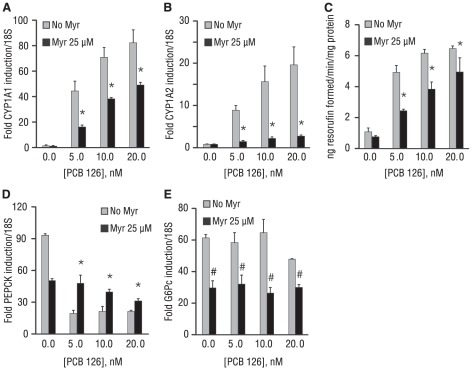
Validation and evaluation of myricetin, a putative competitive AhR inhibitor. Doses of PCB 126 were selected based on the concentrations near the middle (5 nM) or top (10–20 nM) of the respective dose-response curves (established in figure 4). (A) Maximally effective myricetin doses (25 µM) significantly antagonize PCB 126 –induced CYP1A1 gene expression. (B) Similar effect of 25 µM myricetin on PCB 126 induction of CYP1A2 gene expression. (C) 25 µM myricetin antagonizes EROD activity induction by PCB 126. (D) Myricetin homogeneously suppresses forskolin-induced PEPCK expression and also restores PEPCK gene induction by forskolin in the presence of PCB 126. (E) Myricetin homogeneously suppresses forskolin-induced G6pc gene induction by PCB 126, which has no impact on forskolin induction of G6pc expression. (Panels A–C) *p<0.05 for myricetin effect with respective PCB 126 concentration. (Panels D–E) #p<0.05 for myricetin effect within each [PCB 126]. (All panels) Data are average of 2–3 experiments; error bars are −/+ SD.

To evaluate, directly, involvement of the AhR in suppression of PEPCK by PCB 126, AhR inhibitors were screened against 5, 10, or 20 nM PCB 126; these doses were selected based upon the observed relationship between AhR activation and PEPCK suppression (**Figure 4A–C**). An inhibitor-based approach was chosen due to the very limited practical (functional) utility of mature primary hepatocytes in culture, which precluded an RNA interference approach in this study (see Discussion for more details). After screening over one dozen compounds shown (in the literature) to antagonize AhR, only a single flavonol compound, myricetin, [36], was able to antagonize AhR according to pre-established screening parameters. That is, at the effective dose, the compound must (a) not be toxic (**Figure S4**), (b) antagonize AhR as assessed by CYP1A1 (**Figure 5A**), CYP1A2 (**Figure 5B**), and EROD (**Figure 5C**), and (c) have as little intrinsic impact on basal or forskolin-stimulated PEPCK and G6pc as possible. Although myricetin significantly suppressed forskolin-stimulated gluconeogenic gene expression (**Figures 5D and 5E**), the effect was uniform across treatment parameters for both PEPCK and G6pc; myricetin also possessed the most favorable ratio of AhR suppression versus impact on PEPCK and G6pc gene induction amongst the candidate inhibitors. More importantly, myricetin largely restored forskolin-stimulated PEPCK expression when co-incubated with maximally effective concentrations of PCB 126 (5–20 nM; *effective* defined on the basis of AhR activation), restoring PEPCK expression to levels seen in the presence of myricetin+forskolin alone. In contrast, the effect of PCB 126 exposure on G6pc expression in the absence and presence of myricetin was negligible (**Figure 5E**).

Myricetin was maximally effective at 25 µM, although 10 µM of myricetin significantly antagonized low (5 nM) concentrations of PCB 126 (**Figure S5**). At both 10 µM and 25 µM doses, myricetin' s antagonizing power (i.e. the ability of myricetin to reverse PCB 126 suppression of forskolin-induced PEPCK mRNA and antagonize PCB 126 induction of AhR) decreased as the ratio of PCB 126: myricetin increased (Figure S5). Myricetin was not toxic at the maximally effective dose of 25 µM either independently or in conjunction with the highest dose of PCB 126 against which it was concurrently tested (100 nM PCB 126; Figure S4).

### Other non ortho-substituted dioxin-like PCBs have similar effects as PCB 126 on PEPCK

We subsequently tested the hypothesis that AhR activation by other dioxin-like PCBs would suppress forskolin-stimulated expression of PEPCK, but not G6pc, in a manner similar to PCB 126. In addition to PCB 126, three other non-ortho-substituted coplanar dioxin-like PCBs were selected for testing: PCB 169, PCB 81, and PCB 77 (**Table 1**). PCB 105 was included to represent a mono-ortho-substituted coplanar dioxin-like PCB with a significantly lower TEF relative to the other dioxin-like PCBs [8], [37]. PCB 153 served as a negative PCB control, as it is both non-coplanar and non-dioxin-like, and as such, has no TEF [8]. The PCBs were tested at five times lower and higher doses (20 nM and 500 nM, respectively) compared to the commonly used dose of PCB 126, 100 nM. The decision to use higher starting concentrations of PCBs 169, 81, and 77 (i.e. 20 nM instead of 0.05 nM) was based upon the significantly lower TEFs of these PCBs versus PCB 126. The treatment protocol was identical to the standard regimen used throughout this paper (16 h).

**Figure 6:**
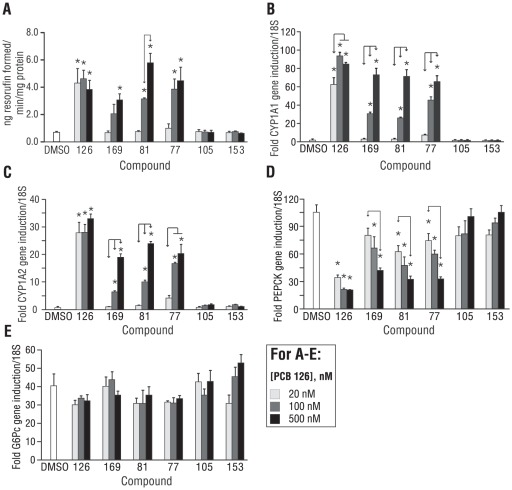
Other dioxin-like PCBs that activate AhR suppress PEPCK gene induction. (A) Dioxin-like PCBs 126, 169, 81, and 77 induce EROD activity, with dose-dependency for all dioxin-like PCBs with the exception of PCB 126. (B) Similar trends observed for when CYP1A1 was used as the readout instead of EROD. (C) CYP1A2 gene expression demonstrates a pattern in line with observations made in panels A and B. (D) Forskolin-induced PEPCK gene expression is suppressed by other dioxin-like PCBs in a dose-dependent fashion, with the exception of PCB 126. (E) G6pc gene induction is not affected by any tested PCBs at any tested concentration. *p<0.05 vs. DMSO, significant differences between doses of PCBs are designated by arrows. Note: for clarity, the *PCB* prefix has been eliminated for the tested congeners on the x-axis label. Data are average of 2–3 experiments; error bars are −/+ SD.

AhR activation was assessed by the EROD assay (**Figure 6A**), as well as quantification of CYP1A1 (**Figure 6B**) and CYP1A2 (**Figure 6C**) gene expression. With the exception of PCB 105, all of the dioxin-like PCBs were potent activators of the AhR, while, as expected, PCB 153 was ineffective as an AhR agonist. At the lowest tested dose for the PCBs (20 nM), only PCB 126 induced CYP1A activity and gene transcription in a statistically significant manner (**Figures 6A–C**). PCB 126 maximally induced CYP1A1 and CYP1A2 at 100 nM, with no further increase at 500 nM; the other dioxin-like PCBs clustered together with respect to their potency towards CYP1A1 induction, with statistically significant dose-dependence, (**Figure 6B**). At each respective low, middle, and high dose, PCB 169, PCB 81 and PCB 77 induced CYP1A1 to levels that were not significantly different between compounds (**Figure 6B**). Similar trends were observed for CYP1A2 (**Figure 6C**).

The pattern of EROD activity closely paralleled that of CYP1A1 gene transcription, with a few notable differences. First, the greatest overall induction of EROD was observed with 500 nM PCB 81 as opposed to any dose of PCB 126. Also, EROD activation by PCB 169 was notably weaker than that of the other dioxin-like PCBs, despite it having the second highest TEF of the dioxin-like PCBs screened and a relative AhR binding potency equivalent to that of PCB 81 [37].

The effects of the various PCBs on forskolin-stimulated PEPCK (**Figure 6D**) and G6pc (**Figure 6E**) gene expression were then evaluated. At the lowest tested dose for this set of experiments (20 nM), PCB 126 was maximally effective at suppressing forskolin-induced PEPCK; there was no additional statistically significant decrease at 100 nM or 500 nM. For all of the other dioxin-like PCBs, there was a dose-response with respect to PCB concentration and PEPCK gene suppression. For suppression of forskolin-induced PEPCK gene expression, there were statistically significant dose effects both within and between compounds at the two lower concentrations of each dioxin-like PCB (excluding PCB 126). However, at 500 nM, all of the non-ortho-substituted dioxin-like PCBs maximally reduced forskolin-stimulated PEPCK expression, such that there was no significant difference amongst the dioxin-like PCBs, including PCB 126. Consistent with 100 nM PCB 126, none of the PCBs, at any concentration, affected forskolin-stimulated G6pc gene expression (**Figure 6E**). When the effect of the various PCBs (at 20, 100, and 500 nM) on magnitude of CYP1A1 induction was plotted magnitude of suppression of forskolin-stimulated PEPCK expression, a statistically significant interaction was observed (**Figure S6**). When CYP1A2 or EROD was substituted for CYP1A1, a similar, statistically significant interaction was seen (data not shown).

## Discussion

TCDD, due to its extreme toxicity compared other dioxins and dioxin-like compounds, is the most widely utilized compound for dioxin-exposure investigations. Although the TEF of PCB 126 is an order of magnitude lower than that of TCDD, widespread production of PCBs for over sixty years implies that the distribution and concentration of PCB 126, as well as other dioxin-like PCBs, may be more penetrant than that of TCDD. One of the few published in vivo metabolic studies using dioxin-like PCBs reported (Clophen A50, a technical PCB mixture whose TEF is primarily attributable to PCB 126 [10]), a similar hypoglycemic effect in PCB-treated rats [21]. However, while in vivo studies afford insight into whole-body effects, *in vitro* studies allow for resolution of direct versus indirect effects. Our findings support the conclusion that PCB 126 and other non-ortho-substituted dioxin-like PCB congeners directly suppress hepatic GNG, an effect mediated through specific suppression of PEPCK gene transcription by a mechanism that involves the AhR.

Others have reported depression of total hepatic glycogen following TCDD exposure in rats [9], [11] mice [38], fish, [39] and more recently in embryonic chick liver [34]. However, studies focused on the effects of PCB 126 on liver glycogen in mammals are lacking. Furthermore, the majority of studies using TCDD or PCB mixtures observed decreased glycogen following a several-day period of treatment [9], [11], or used doses that induced nonspecific toxicity [40]. The fact that short-term treatment with PCB 126 was able to directly suppress hepatic glycogen —independent of cytotoxicity—implies that impaired glycogen metabolism may play an early and primary role in dioxin-induced hypoglycemia, as opposed to being a secondary, nonspecific phenomenon. The mechanisms by which PCB 126 suppresses glycogen remain unclear, as PCB 126 did not affect basal GNG gene expression or unstimulated rates of glucose production, nor were changes in the level or phosphorylation state of glycogen synthase and phosphorylase detected (data not shown). Non-genomic signaling or allosteric enzyme regulation may each play a role, given that maximum suppression of glycogen required concentrations of PCB 126 in excess of the dose needed for maximal AhR activation. In cell lines, TCDD, through a non-AhR-mediated mechanism, induces release of intracellular calcium [41], which may accelerate glycogenolysis; the lack of effect of PCB 126 on glycogenolytic rates in our system implies that this is either not the mechanism, or that experimental conditions may require modification to specifically interrogate this pathway.

The contribution of GNG to long-term maintenance of euglycemia during fasting vastly surpasses that of glycogen (reviewed in [42]). TCDD and PCB 126 suppress both of the two principal, hormonally regulated gluconeogenic genes, PEPCK and G6pc, in vivo and *in vitro*. However, in mammalian studies, PEPCK suppression occurs earlier and to a greater extent than G6pc, and demonstrates dose-responsiveness—implying that in mammalian liver, PEPCK suppression may be specific, and G6pc suppression a secondary effect.

**Figure 7:**
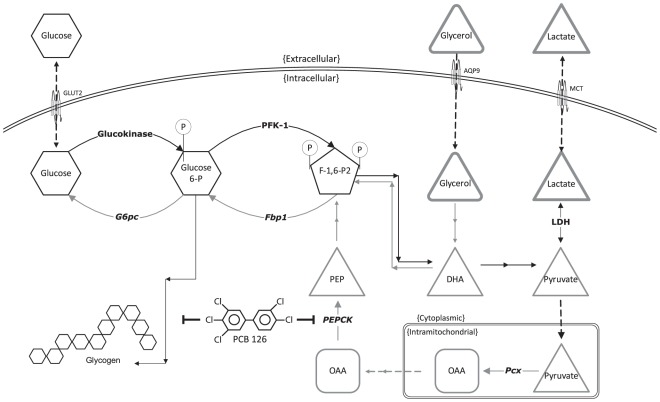
Simplified visual model of the impact of PCB 126 on hepatic glucose metabolism. Glycolysis, glycogen synthesis, and gluconeogenesis in a mouse hepatocyte are presented. Forward metabolism of glucose (glycolysis and glycogen synthesis) is depicted by black arrows, while reverse metabolism of glucose (GNG) is denoted by gray arrows. Enzymes involved in forward glucose metabolism are in standard bold text, while enzymes involved in reverse glucose metabolism are in italicized bold text. In this model, PCB 126 inhibits GNG from lactate (and pyruvate) by suppression of PEPCK gene transcription, and also inhibits glycogen by an unknown mechanism. GNG from glycerol is not affected by PCB 126 because glycerol is under the control of Fbp1 and G6pc under conditions that promote GNG, and thus bypasses PEPCK.

Both TCDD and a PCB mixture suppress the upregulation of PEPCK induced by fasting or cAMP analogs [11], [21], making the induced condition (forskolin-stimulated in our study) potentially more physiologically relevant. Furthermore, we tested lactate and glycerol as gluconeogenic substrates based on their divergent points of entry in the gluconeogenic pathway (**Figure 7**). In mammals, synthesis of glucose from lactate is under the control of several critical gluconeogenic enzymes, including Pcx, cytosolic PEPCK, Fbp1, and G6pc. In contrast, glycerol enters the gluconeogenic pathway at a more proximal point, and, enzymatically, is subject primarily to regulation by Fbp1 and G6pc [43]. Using these substrates under unstimulated and stimulated conditions, we found that PCB 126 selectively suppresses forskolin-stimulated GNG from lactate, but not glycerol. During gluconeogenesis, pyruvate is regulated in a manner similar to lactate in the rodent, and we observed similar patterns when substituting pyruvate for lactate. However, in our hands, pyruvate (compared to lactate) yields a significantly lower forskolin effect in the GNG assay, such that statistically significant resolution of PCB 126's effect is impractical. The underlying reasons for this discrepancy *in vitro* are unknown, but may be related to lactate's provision of reducing power (NADH), a requirement for sustaining high (i.e. forskolin-induced) rates of GNG. Our combined metabolic and molecular findings demonstrate that PCB 126 selectively influences forskolin-stimulated expression of PEPCK. The absence of effect of PCB 126 on either Pcx or Fbp1 lends additional support to the specificity of PCB 126 in GNG.

Although previous studies hypothesize an AhR-independent mechanism for dioxin-mediated PEPCK suppression [20], [44], AhR activation is the prototypical mechanism of action for TCDD and the dioxin-like PCBs. The current data, in contrast to previous findings, support direct involvement of the AhR. First, PCB 126 dose-dependently suppressed forskolin-stimulated PEPCK expression, and the concentration of PCB 126 required for this effect correlated with the magnitude of AhR activation, demonstrated by the dose-response curves for CYP1A1 and EROD. Second, myricetin, an AhR antagonist, largely restored PCB 126-induced suppression of PEPCK, and this reversal was ablated at doses of PCB 126 high enough (>20 nM) to overcome the inhibitory effect of myricetin with respect to the AhR. Third, other non-ortho-substituted dioxin-like PCBs similarly suppressed forskolin-induced PEPCK expression in a dose-dependent manner. Finally, a recent publication reported that in chicken embryo hepatocytes, AhR mediates TCDD suppression of GNG and gluconeogenic genes, but through an indirect mechanism involving the TCDD-inducible PARP (TiPARP) [34]. The findings of this study differ from ours in that both PEPCK and G6pc were suppressed by TCDD; differences in the compound used (TCDD vs. PCB 126) as well as the choice of species/developmental stage of the animal may account for these differences. For example, in adult avian species, the mitochondrial isoform comprises up to 90% of total PEPCK, while in the mouse and rat, the majority of PEPCK is cytosolic; in humans, the two PEPCK isoforms are roughly equally distributed (reviewed in [42]).

Gene manipulation and/or more specific, potent AhR inhibitors are potentially useful tools for further elucidation of mechanism. The former would be ideal, but primary hepatocytes in standard two-dimensional culture are highly dynamic, and their phenotype changes rapidly over the course of hours, with corresponding alterations in gene expression and functional parameters [45]. Metabolic endpoints in particular rapidly fall below the limits of reliable detection with available quantitation methods; variance in absolute substrate output and percent hormone response increases substantially as well (own observations and communications).

We are unaware of existing published studies that couple a diverse array of functional readouts (hormone-responsive glycogen synthesis, GNG, lipogenesis, and beta-oxidation) with classic molecular (gene expression) and biochemical (EROD) methods in the field of toxicology. The relative lack of studies using a direct *in vitro* knockdown approach to evaluate similar metabolism-based endpoints in primary hepatocytes perhaps highlights the associated challenges. Recently, Mao et al. (2011) investigated the effects of IRE1α knockdown in the liver [46]. The authors transfected primary hepatocytes *in vitro* with the IRE1α construct for immunoblotting and measured GNG in primary mouse hepatocytes by a method similar to ours. Notably, the effect of IRE1α siRNA on GNG (*in vitro*) was performed in hepatocytes isolated from mice injected with virus beforehand. Thus, primary hepatocytes are readily amenable to molecular manipulation post-plating, but functional *in vitro* functions may be practical only within a very short window using existing two-dimensional culture techniques.

Pharmacological AhR antagonists present an alternate or supplemental strategy for mechanistic AhR investigations in primary hepatocytes. Unfortunately, there is no widely available pharmacological inhibitor for the AhR at the moment. Many studies have reported the ability of plant-derived flavones, flavonols, and related compounds to inhibit the AhR. Our inhibitor screen of over one dozen candidates found minimal efficacy with several relatively well-characterized compounds even at high micromolar concentrations (i.e. alpha-naphthoflavone, resveratrol, and quercetin), while other putative inhibitors (i.e. curcumin and apigenin) had highly variable mouse-to-mouse efficacy. Almost all tested inhibitors that suppressed AhR activation by PCB 126 interfered with forskolin-stimulated GNG gene expression as well. Even myricetin, the most efficacious candidate, had moderate (but homogeneous) effects on forskolin-stimulated PEPCK and G6Pc expression. Finally, certain inhibitors suppressed EROD activity induction by PCB 126 but augmented CYP1A1 and CYP1A2 gene transcription (i.e. α- naphthoflavone), or suppressed CYP1A1 and CYP1A2 gene expression but upregulated EROD activity (i.e. danthron). These seemingly contradictory observations emphasize the importance of evaluating both EROD and an AhR target gene (or genes) during inhibitor screens to differentiate between direct effects on EROD enzymatic activity versus true AhR antagonism.

The mechanism by which productive AhR binding leads to inhibition of PEPCK gene transcription is currently unclear. The core AhR response element is present in the region 5 KB upstream of the mouse cytosolic PEPCK transcription start site, and Tijet et al. (2005) reported that the mouse PEPCK gene contains 7 core AhREs in the −5 KB to +1 KB region [47]. In their study, a single high-dose TCDD injection in mice (19 h induction period) resulted in a 4-fold decrease in liver PEPCK expression in wild-type mice; no impact of TCDD was observed in AhR-knockout mice. Our findings are in agreement with this and previous reports, additionally emphasizes the specificity of this effect towards the gluconeogenic pathway, and most importantly, bridges existing *in vitro* molecular and biochemical findings with in vivo observations by providing functional *in vitro* data (i.e. GNG). In-depth mechanistic studies will be required to elucidate the mechanism by which AhR activation inhibits PEPCK expression.

PCB-mediated inhibition of gluconeogenesis potentially threatens glucose homeostasis and energy metabolism in exposed humans and wildlife. In apex predators known to bioaccumulate PCBs (e.g. polar bears), high levels of dioxin-like PCBs may impair gluconeogenesis during periods of fasting. This could be particularly dangerous during prolonged periods of food restriction, e.g. during periods of hibernation or when food availability is otherwise restricted. In humans, PCB-induced disruptions in gluconeogenesis may promote hypoglycemia in susceptible patients during fasting, an effect potentially magnified by commonly coincident impairments in hepatic function seen in disease conditions presenting with hypoglycemia (e.g. non-alcoholic fatty liver disease, hepatitis, alcohol abuse). Impairments in hepatic glucose mobilization may be particularly important in individuals taking anti-diabetic medications that predispose the subject to hypoglycemia (e.g. sulfonylureas, insulin). Even brief and remote periods of hypoglycemia can lead to lasting impairments in cognitive function and potentially life threatening situations. Thus, agents that inhibit escape from hypoglycemia may pose significant long-term health risks. Given the environmental persistence of dioxin-like PCBs, these chemicals may be overlooked, underappreciated disruptors of energy metabolism.

In conclusion, this study presents the following novel findings: (a) PCB 126 directly and rapidly suppresses hepatic glycogen metabolism; (b) PCB 126 selectively and potently suppresses forskolin-stimulated, but not basal GNG from lactate, but not glycerol; (c) PCB 126 selectively, dose-dependently, and directly suppresses forskolin-stimulated, but not basal PEPCK expression and has no effect on the other key gluconeogenic genes; (d) myricetin antagonizes AhR and largely restores forskolin-stimulated PEPCK expression; (e) other dioxin-like PCBs that productively bind the AhR mimic the effect of PCB 126 on forskolin-stimulated PEPCK gene expression. Future investigations using direct AhR and/or ARNT knockdown, comparison of poorly versus readily metabolizable AhR ligands on GNG/PEPCK, and expanding the database of dioxins and dioxin-like compounds with respect to their effect on hepatic GNG and gluconeogenic genes may offer additional mechanistic insight.

## Supporting Information

Figure S1 **Dioxin-like PCBs suppress glycogen, while non-dioxin-like PCBs do not.** PCB 77 tended to suppress glycogen, but this did not consistently reach statistical significance (p<0.10); *p<0.05 vs. DMSO. Data are average of 2–3 experiments; error bars are −/+ SD.
(EPS)

Figure S2 **PCB 126 does not affect total intracellular ATP content.** Total intracellular ATP is not impacted by PCB 126 (100 nM) after incubation in 5 mM or 10 mM glucose-containing serum-free medium. Data are average of 2 experiments; error bars are −/+ SD. Note that the effect of glucose on total intracellular ATP tended towards statistical significance (p<0.10).
(EPS)

Figure S3 **PCB 126 does not allosterically affect GNG from lactate.** Addition of PCB 126 only at the onset of the GNG assay, and maintaining its presence throughout the standard four-hour collection period did result in suppression of forskolin-stimulated GNG from lactate. Note that data are the sum of four independent samples collected at one-hour intervals; *p<0.05 for forskolin effect relative to respective −/+ PCB condition. Data are average of 2–3 experiments; error bars are −/+ SD.
(EPS)

Figure S4 **Myricetin is not cytotoxic in the presence or absence of 100 nM PCB 126.** At the maximally effective dose of 25 µM, myricetin does not increase intracellular LDH release nor impact total LDH activity, independently or in conjunction with PCB 126 (100 nM). Other final-round candidate inhibitors are additionally presented for context. Data are average of 2–3 experiments; error bars are −/+ SD.
(EPS)

Figure S5 **Myricetin dose-dependently suppresses AhR induction, and this effect wanes as [PCB 126] increases.** A complete dose-response curve for PCB 126 is presented, although myricetin (10 µM and 25 µM) was only tested against pre-determined concentrations of PCB 126 (0, 5 nM, 10 nM, and 20 nM; 25 µM myricetin was always tested against 100 nM PCB 126 as a negative control for myricetin efficacy. *p<0.05 for myricetin effect vs. respective no-myricetin PCB condition. Data are average of 2–3 experiments; error bars are −/+ SD.
(EPS)

Figure S6 **Standard least squares analysis of CYP1A1 activation versus PEPCK induction.** PCBs 126, 169, 81, and 77 at 20, 100, and 500 nM were compared with respect to their activation of CYP1A1 gene expression (x-axis) and inhibition of PEPCK gene induction (y-axis). Standard least squares linear regression revealed statistically significant relationship between magnitude of CYP1A1 induction and PEPCK suppression (p<0.05). Data are derived from experiments performed for figure 6. Error bars have been excluded for clarity.
(EPS)

Table S1 **qRT-PCR primer sequences and expected amplicon lengths.**
(DOCX)
