# Supplementary material for: PCB 126 and Other Dioxin-Like PCBs Specifically Suppress Hepatic PEPCK Expression via the Aryl Hydrocarbon Receptor
Source: PLoS One. 2012 May 16;7(5):e37103. doi: 10.1371/journal.pone.0037103 (PMC3353882; doi:10.1371/journal.pone.0037103)
Supplement: Table S1 — qRT-PCR primer sequences and expected amplicon lengths. (DOCX) [file pone.0037103.s007.docx]

| **Gene** | **RefSeq** | **Direction** | **Primer sequence; 5' → 3'** | **Amplicon size, bp** |
| --- | --- | --- | --- | --- |
| 18S | NR_003278.1 | Forward | CGGCTACCACATCCAAGGA | 187 |
|  |  | Reverse | GCTGGAATTACCGCGGCT |  |
|  |  |  |  |  |
| CYP1A1 | NM_009992.3 | Forward | TATCTCGTCAGCAAACTTCAG | 100 |
|  |  | Reverse | ATATGGCACAGATGACATTGG |  |
|  |  |  |  |  |
| CYP1A2 | NM_009993.3 | Forward | ACCGATACACATCCTTTGTC | 131 |
|  |  | Reverse | CTTCTCATCATGGTTGACCT |  |
|  |  |  |  |  |
| Fbp1 | NM_019395.2 | Forward | TCGCACAGCTCTATGGTATCG | 124 |
|  |  | Reverse | AGAACACAGGTAGCGTAGGAC |  |
|  |  |  |  |  |
| G6pc | NM_008061.3 | Forward | TGCAAGGGAGAACTCAGCAA | 145 |
|  |  | Reverse | TTGCGCTCTTGCAGAAAGAC |  |
|  |  |  |  |  |
| PEPCK | NM_011044.2 | Forward | TGGTGGGAACTCACTACTCGG | 105 |
|  |  | Reverse | ATGCCCAGGATCAGCATATGC |  |
|  |  |  |  |  |
| Pcx | NM_001162946.1 | Forward | TGCCAAGCAGGTAGGCTATGA | 91 |
|  |  | Reverse | GCGGGAATTGACCTCGATGAA |  |
